# The Mpox Vaccine Hesitancy Scale for Mpox: Links with Vaccination Intention among Men Who Have Sex with Men in Six Cities of China

**DOI:** 10.3390/vaccines12091009

**Published:** 2024-09-03

**Authors:** Ying Gao, Shangbin Liu, Huifang Xu, Ying Wang, Gang Xu, Fan Hu, Jiechen Zhang, Yong Cai

**Affiliations:** 1 Public Health Department, Hongqiao International Institute of Medicine, Tongren Hospital, Shanghai Jiao Tong University School of Medicine, Shanghai 200025, China; gy200217@sjtu.edu.cn (Y.G.); liushangbin@sjtu.edu.cn (S.L.); huifangxu@sjtu.edu.cn (H.X.); yingwangxun@sjtu.edu.cn (Y.W.); hufan@sjtu.edu.cn (F.H.); 2 School of Public Health, Shanghai Jiao Tong University School of Medicine, Shanghai 200025, China; gangxu@sjtu.edu.cn; 3 Dermatology Department, Tongren Hospital, Shanghai Jiao Tong University School of Medicine, Shanghai 200025, China

**Keywords:** Mpox, vaccination intention, men who have sex with men, protection motivation theory, validity, reliability

## Abstract

Background: Vaccine hesitancy is a significant barrier to achieving high vaccination rates, particularly among men who have sex with men (MSM), a group at increased risk for Mpox. This study aimed to develop and validate a Mpox vaccine hesitancy scale specifically tailored for Chinese MSM, grounded in the protection motivation theory (PMT). Methods: An online survey through snowball sampling was conducted from October 2023 to March 2024, collecting 2403 valid responses across six representative regions in China. Exploratory factor analysis (EFA) and confirmatory factor analysis (CFA) were conducted to evaluate the scale’s construct validity, while reliability was assessed using Cronbach’s α coefficient. The predictive validity of the scale was analyzed using Receiver Operating Characteristic (ROC) analysis. Results: EFA ultimately retained 22 items in the Mpox vaccination scale and identified four distinct dimensions: Maladaptive Rewards (seven items), Self-efficacy (seven items), Response Efficacy (four items), and Response Costs (four items). These dimensions were confirmed by CFA, which demonstrated satisfactory model fit indices (χ²/df = 4.382, RMSEA = 0.053, SRMR = 0.048, GFI = 0.935, CFI = 0.967, NFI = 0.958, TLI = 0.963, and IFI =0.967). All indices were within acceptable ranges. The scale exhibited good internal consistency, with a Cronbach’s alpha of 0.906, and strong content validity, with an S-CVI/Ave of 0.952. The scale’s predictive accuracy was evaluated using Receiver Operating Characteristic (ROC) analysis. When used to evaluate the scale’s predictive accuracy, it yielded an area under the curve (AUC) of 0.854 after adjustments, indicating good predictive ability between high and low hesitancy. Conclusions: This scale provides a reliable and valid tool for assessing Mpox vaccine hesitancy among MSM and can be used to gauge Mpox vaccination intention within this group.

## 1. Introduction

Mpox (monkeypox) is a viral disease caused by the monkeypox virus, a species within the Orthopoxvirus genus. Typical symptoms include fever, severe headache, lymphadenopathy, back pain, rash, and myalgia [1]. Since its initial identification in humans in 1970, Mpox has occurred sporadically in Central and West Africa [2,3]. However, a significant outbreak emerged in May 2022, rapidly spreading worldwide [3,4,5]. China’s first imported case of Mpox was reported in September 2022, and the epidemic began in June 2023, with over 20 provinces reporting cases [6]. As of 31 May 2024, 97,208 confirmed Mpox cases and 186 related deaths had been reported across 117 countries [7]. The World Health Organization (WHO) declared the Mpox outbreak a Public Health Emergency of International Concern (PHEIC) twice, first in July 2022 and again in August 2024 [8].

Although anyone can contract Mpox, the 2022 outbreak predominantly spread through sexual networks, with nearly all cases occurring among men who have sex with men (MSM), including gay and bisexual men [9,10,11]. This transmission pattern is likely related to the nature of sexual contact among MSM, such as anal intercourse and oral sex, which involve direct mucosal contact, thereby facilitating viral transmission [12]. Moreover, studies suggest that MSM may exhibit different immune statuses, including higher rates of human immunodeficiency virus (HIV) infection, potentially making them more susceptible to Mpox [13].

The WHO maintains that vaccination against Mpox, combined with avoiding high-risk contact with infected individuals, constitutes an effective preventive strategy [14]. Vaccination is a cornerstone of public health, dramatically reducing the incidence of numerous vaccine-preventable diseases [15,16]. Widespread vaccination is not recommended at this time; only individuals who have been in close contact with Mpox patients or those at high risk of exposure should consider receiving the vaccine [14]. However, a meta-analysis of 29 studies revealed that the global prevalence of intention to vaccinate against Mpox was only 61%, and the aggregated prevalence of refusal to vaccinate reached 22%, indicating a significant level of vaccine hesitancy [17]. Understanding the factors that influence vaccination intentions and behaviors is critical to increasing Mpox vaccine uptake and achieving optimal levels of disease prevention [17,18].

Existing research suggests that vaccination willingness is shaped by a combination of factors, including sociodemographic variables, psychological factors, beliefs about Mpox, and vaccine confidence [4,19,20,21]. However, current research on Mpox vaccination intention lacks a strong theoretical foundation. Protection motivation theory (PMT) posits that cognitive processes regulate the relationship between attitudes and behavior change, emphasizing that individuals’ motivation, driven by cognitive appraisal processes, can lead to protective behaviors against perceived threats [22,23]. PMT has been widely applied in the interpretation, prediction, and intervention of health behaviors, including vaccination uptake [24,25,26], smoking [27], and cancer screening [28]. Therefore, this study aimed to develop a Mpox vaccine hesitancy scale based on the protection motivation theory and verify its reliability and validity in Chinese MSM. By providing a reliable and valid measurement tool, this study provides appropriate measurement tools for future studies and helps explore strategies to improve vaccine uptake in this high-risk population.

## 2. Methods

### 2.1. Development of the Scale

The complete online questionnaire included sections on general sociodemographic information, sexual behavior-related information, lifestyle factors, disease history, Mpox diagnosis history, and the Mpox vaccine hesitancy scale.

In developing the scale and questionnaire, the research team sought suggestions from experts in dermatology, public health, infectious disease, management, and NGOs. The suggestions were independent of their official duties. Additionally, team members reviewed several studies grounded in protection motivation theory [29,30,31]. This process resulted in an item pool comprising 24 items and the formulation of four scale dimensions: Maladaptive Rewards (7 items), Self-efficacy (7 items), Response Efficacy (4 items), and Response Costs (6 items). The scale employed a 5-point Likert scoring system, with the scores for the Maladaptive Rewards and Response Costs dimensions being positively scored, while the scores for the Self-efficacy and Response Efficacy dimensions were reverse-scored. Higher scores indicated greater hesitancy towards initiating the free Mpox vaccine.

Prior to administering the full survey, cognitive interviews were conducted with a small group of MSM participants as a pre-test. These interviews aimed to assess the clarity and comprehensibility of the questions, ensuring that participants were able to interpret them correctly. Based on the feedback received during these interviews, minor revisions were made to the wording of certain questions to enhance their clarity.

### 2.2. Participants

With the assistance of non-governmental organizations (NGOs) in participant recruitment, an online anonymous cross-sectional survey was conducted using the Wenjuanxing (Changsha Ranxing Information Technology Co., Ltd., Changsha, China) platform between October 2023 and March 2024. The survey was carried out simultaneously across six representative geographical regions in China: northwest, southwest, southeast, east, northeast, and central.

Due to the hidden nature of our targeted population, preventing the feasibility of conducting random sampling, convenience sampling and snowball sampling were employed for sample collection. Initially, local non-governmental organizations (NGOs) in each region recruited 5–10 eligible MSM “seeds” who possessed extensive social networks within the community. These seeds subsequently recruited or referred additional eligible participants, following a chain-referral process until the target sample size was achieved. The sample size required for this study was estimated based on the expected prevalence of Mpox in MSM, which was determined to be 0.73% according to existing research [32]. The specific formula is detailed as follows.

$$N=\frac{Z^{2}\cdot P\cdot (1-P)}{E^{2}}$$

*P* represents the proportion of the MSM expected to have Mpox, which was estimated to be 0.73% according to an existing study. For a 95% confidence level, the *Z*-value used was 1.96. A margin of error (*E*) of 0.5% was used to ensure precise estimates.

**Figure 1:**
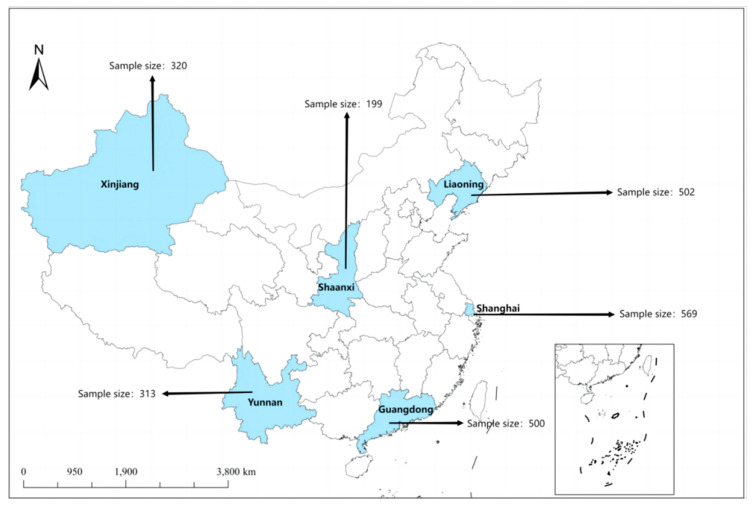
Source of participants. The diagram illustrates study participants from six representative cities, highlighting the total number of 2403 samples recruited and the final sample size used for analysis.

Assuming simple random sampling, the formula indicated the need for 1114 participants. However, given that snowball sampling was employed due to the nature of the target population, an adjustment for the design effect was necessary. Referring to general estimates for complex sampling methods, a design effect (DE) of 2.0 was applied to account for the increased variability associated with snowball sampling. This adjustment resulted in an increased sample size requirement of 2228 participants. To further account for an anticipated 10% non-response rate, the final required sample size was calculated to be 2476 participants. This sample size was deemed sufficient to provide reliable estimates of the prevalence while considering the potential clustering effects and non-response within the snowball sampling framework. Finally, a total of 2481 questionnaires were collected, with 2403 valid responses, yielding an effective response rate of 96.86%. The number of valid questionnaires from each region, as shown in Figure 1, was as follows: Shanghai (569), Guangdong (500), Xinjiang (320), Shaanxi (199), Yunnan (313), and Liaoning (502).

Inclusion criteria were as follows: (1) cisgender male individuals aged 18 years or older; (2) those who had engaged in sexual activities with men within the past six months; and (3) permanent residents of the selected provinces, municipalities, or autonomous regions, defined as residing locally for the majority of the preceding six months. Exclusion criteria were as follows: (1) participants who completed the questionnaire in under 300 s; (2) responses that failed quality control questions; and (3) IP addresses indicating locations outside the designated geographical regions.

Ethical approval for this study was granted by the Shanghai University of Medicine and Health Sciences (approval number: 2023-MSMMPOX-22-310222197604080237). Informed consent was obtained from all participants.

### 2.3. Data Collection and Quality Control

The research team provided uniform training to NGO staff, who were the primary investigators across different regions, on survey administration procedures. Trained investigators assisted participants in completing the online survey, including technical support and necessary and non-inducible semantic assistance. The staff ensured participants’ privacy during the survey answering process. Quality control questions were also embedded in the survey to ensure the reliability of the responses.

At the survey sites, staff informed all eligible MSM participants of the following: (1) the survey was conducted anonymously, and all collected data would be kept strictly confidential and used solely for research purposes; (2) refusal to participate would not affect their access to any services provided by the NGO; and (3) participants could withdraw from the study at any time.

Additionally, a data quality supervision team was established to regularly (within 48 h) review and provide feedback on the collected data. After data collection, data processing was undertaken, including checking for outliers, missing data, and logical errors, followed by necessary corrections and adjustments.

### 2.4. Statistical Analysis

All analyses were conducted using IBM SPSS version 22.0 and AMOS 28.0 software, with the significance level set at 0.05. Continuous variables were presented as mean ± standard deviation (SD), while categorical variables were presented as counts and percentages. Independent samples t-tests and chi-square tests were used to compare differences between the low- and high-intention groups for free Mpox vaccination regarding continuous and categorical variables, respectively.

The critical ratio method and correlation coefficient method were applied to select the scale items. First, participants were sorted based on their total scale scores from highest to lowest. The top 27% of scores constituted the high-score group, and the bottom 27% constituted the low-score group [33]. Both groups’ scores conformed to a normal distribution. An independent samples t-test was then conducted between these two groups to determine statistical significance; items demonstrating significant differences were retained. Next, Pearson correlation coefficients were calculated to determine the correlation between each item and the total scale score, retaining items with a correlation coefficient (r) greater than 0.40 [30]. Additionally, the Cronbach’s α coefficient of the scale was calculated after the sequential removal of each item to ensure internal consistency.

Seven experts were invited to assess content validity, providing ratings for each item. Based on the experts’ ratings, the item-level content validity index (I-CVI) and the scale-level content validity index/average (S-CVI/Ave) were calculated. Before exploring the construct validity, all samples were randomly divided into two groups using a random number generator: sample 1 for exploratory factor analysis (EFA) (N = 1202) and sample 2 for confirmatory factor analysis (CFA) (N = 1201). For the EFA, the Kaiser–Meyer–Olkin (KMO) test and Bartlett’s test of sphericity were performed to determine the feasibility of factor analysis. Principal component analysis was then conducted to explore the dimensions of the Mpox vaccine hesitancy scale. The CFA was used to investigate the model fit indices of the scale, including the chi-square goodness-of-fit (χ²/df), root mean square error of approximation (RMSEA), standardized root mean square residual (SRMR), goodness-of-fit index (GFI), comparative fit index (CFI), normed fit index (NFI), Tucker–Lewis index (TLI), and incremental fit index (IFI). Generally, a model is considered to have a good fit when χ²/df < 5.00 [34], RMSEA < 0.08, SRMR < 0.05, and GFI, CFI, NFI, TLI, and IFI > 0.90 [33,35]. Combined reliability (CR), average variance extracted (AVE), and the correlation coefficients of the scale dimensions were also calculated. Convergent validity is considered good when CR > 0.70 and AVE > 0.50 [36]. Discriminant validity is acceptable when the correlation coefficients between the scale dimensions are less than the square root of the AVE [36]. Additionally, our study utilized internal consistency and split-half reliability to assess the reliability of the scale. The Cronbach’s α coefficient for the Mpox vaccine hesitancy scale and each of its dimensions was calculated to evaluate internal consistency, with a coefficient of 0.60 typically being considered the minimum acceptable standard for reliability [37,38].

Lastly, Receiver Operating Characteristic (ROC) analysis was conducted to evaluate the predictive ability of the Mpox vaccine hesitancy scale. The analysis involved calculating the area under the curve (AUC) values for the four individual domains, as well as their combination. Adjustments for demographic characteristics, sexual behavior, lifestyle factors, disease history, and Mpox diagnosis history were made to further assess the scale’s predictive accuracy.

## 3. Results

### 3.1. Sample Characteristics

This study finally involved a total of 2403 participants, with 310 expressing low intention to receive the free Mpox vaccine and 2093 showing high intention. The average age of all participants was 30.59 years (SD = 8.02). Among them, 1887 (78.5%) identified as homosexual, 199 (8.3%) were HIV-positive, 221 (9.2%) had sexually transmitted diseases, 1197 (49.8%) had multiple male sexual partners, 1008 (41.9%) frequently engaged in unprotected anal intercourse, and 56 (2.3%) had been diagnosed with Mpox. Other sample characteristics are displayed in Table 1.

### 3.2. Item Analysis

The absolute values of the critical ratios (CRs) for each item were all greater than 3, ranging from 11.123 to 49.141 (all *p* < 0.001). This indicates that the items on the scale have high discriminative power. Additionally, the Pearson correlation coefficients between the scores of each item and the total score of their respective dimensions ranged from 0.637 to 0.905 (all *p* < 0.001), indicating that the items have good representativeness. After individually removing each item, the Cronbach’s α coefficient of the scale ranged from 0.900 to 0.909. In conclusion, all items of the scale were retained (Table 2).

### 3.3. Validity

#### 3.3.1. Content Validity

Seven professionals specializing in dermatology, public health, or infectious disease were invited to assess the content validity. After the evaluation of each item, the I-CVI ranged from 0.857 to 1.000, and the S-CVI/Ave was 0.952. Each item was found to be semantically clear and aligned with its corresponding dimension, manifesting that the questionnaire has good content validity.

#### 3.3.2. Construct Validity

An EFA was conducted on the data from sample 1 (N = 1202). The results indicate a KMO value of 0.923, with Bartlett’s test of sphericity being statistically significant (*χ^2^* = 24,398.547, *p* < 0.001). This suggests that the correlation matrix of the scale data has common factors. Principal component analysis with varimax rotation was further employed, extracting four factors with eigenvalues greater than 1. The variance contributions were 23.659% for factor 1, 21.955% for factor 2, 13.390% for factor 3, and 12.293% for factor 4, resulting in a cumulative variance contribution of 71.314% (Supplementary Table S1).

Items Q19 and Q20, which had factor loadings less than 0.5, were removed. The remaining 22 items were re-analyzed, yielding a KMO value of 0.916 and Bartlett’s test of sphericity *χ^2^* = 23,131.114 (*p* < 0.001). The variance contributions for the four factors were 23.933%, 23.597%, 14.536%, and 11.878%, respectively, with a cumulative variance contribution of 73.943%. The four factors are the same as the structure in the original questionnaire, including seven, seven, four, and four items, respectively, and being labeled as F1-1 to F1-7, F2-1 to F2-7, F3-1 to F3-4, and F4-1 to F4-4. The factor loadings of the items ranged from 0.661 to 0.897 (Table 3).

**Figure 2:**
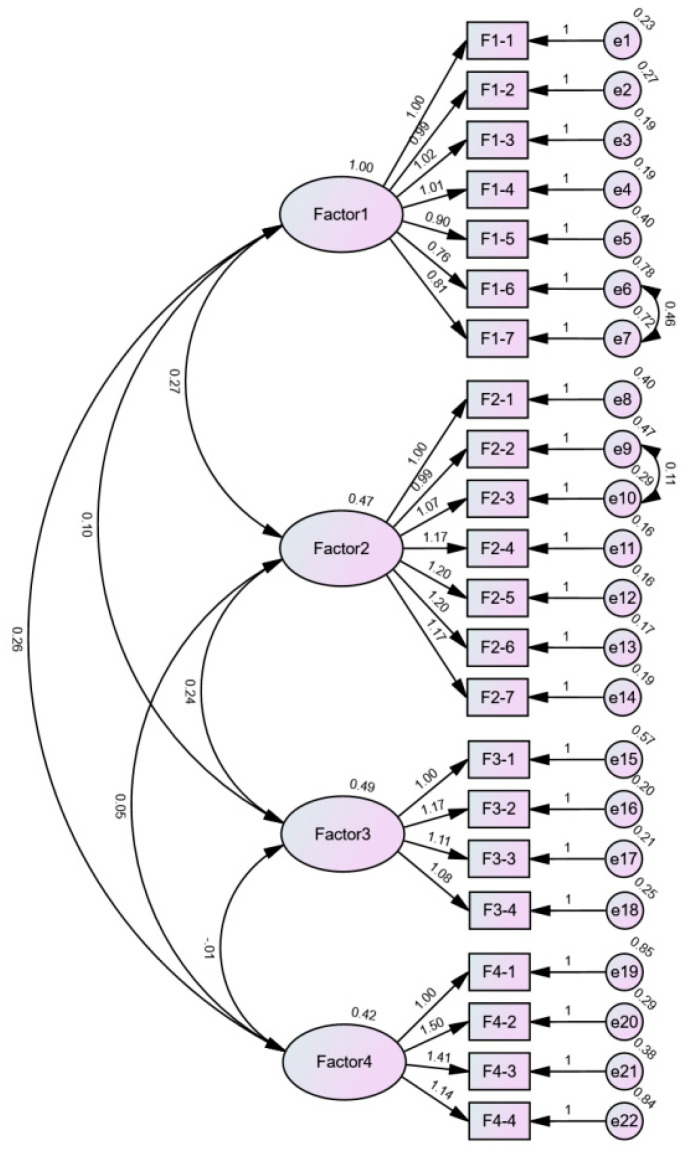
Standardized four-factor structural model of the Chinese Mpox vaccination intention scale (N = 1201). Factor 1: Maladaptive Rewards; Factor 2: Self-efficacy; Factor 3: Response Efficacy; Factor 4: Response Costs.

Subsequently, a CFA was conducted on the data from sample 2 (N = 1201). Amos 28.0 software was used to construct the model and derive the structural equation model (Figure 2). Table 4 presents the goodness-of-fit index. The final complete questionnaire is shown in Supplementary Table S2.

Based on the related calculation formula, the CR values for the four domains of the scale were 0.940, 0.940, 0.889, and 0.821, respectively, all exceeding the 0.70 threshold. The AVE values for each dimension were 0.693, 0.695, 0.670, and 0.543, respectively, all surpassing the 0.50 standard. These results indicate that the convergent validity of the scale meets the criteria. Additionally, the square roots of the AVE values were greater than the corresponding correlation coefficients, demonstrating that the discriminant validity is within an acceptable range (Table 5). Unstandardized and standardized factor loadings are provided in Supplementary Table S3. In conclusion, given all the findings above, the scale demonstrates good construct validity.

### 3.4. Reliability

The Cronbach’s α coefficient for the Chinese vaccination intention scale for men who have sex with men was 0.906, with the Cronbach’s α coefficients for subscales ranging from 0.811 to 0.942. Furthermore, the split-half reliability of the scale was 0.756, with the split-half reliability of each dimension ranging from 0.809 to 0.904 (Supplementary Table S4). In summary, the scale demonstrates good reliability.

### 3.5. ROC Analysis of Predictive Accuracy for Vaccination Intention

**Figure 3:**
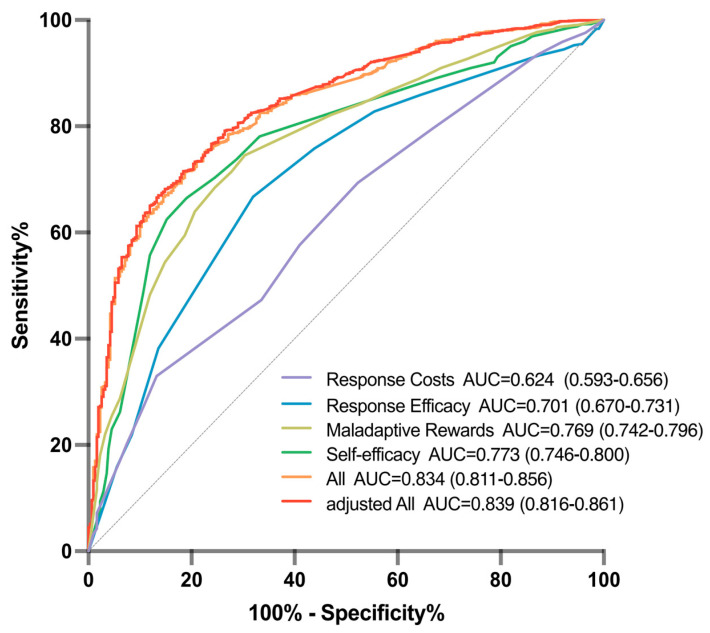
ROC analysis of predictive accuracy for vaccination intention. The Receiver Operating Characteristic (ROC) curve demonstrates the performance of the predictive model for vaccination intention, with the area under the curve (AUC) indicating the model’s accuracy and discriminative ability.

ROC analysis was conducted to evaluate the ability of the scale to predict Mpox vaccination intention among MSM. The AUC values for the four domains were 0.769, 0.773, 0.701, and 0.624, respectively. The combined AUC value for the four domains was 0.834, which is better than the individual domains, indicating relatively good predictive accuracy. After adjusting for demographic characteristics, sexual behavior, lifestyle factors, disease history, and Mpox diagnosis history, the AUC value increased to 0.839, demonstrating the highest predictive accuracy (Figure 3).

## 4. Discussion

Vaccine hesitancy is a phenomenon observed globally, impacting the prevention and control of infectious diseases to varying degrees [39]. Research on vaccine hesitancy in the context of Mpox, especially among high-risk populations such as MSM, has been limited and lacks standardized evaluation tools. Addressing this gap, our study developed and validated the Mpox vaccine hesitancy scale based on PMT, tailored to the cultural and social context of China. This scale demonstrates high reliability and validity, offering a professional tool for assessing vaccine hesitancy that has the potential for global application. Utilizing this scale facilitates a deeper understanding of the factors contributing to vaccine hesitancy among MSM, which can inform targeted interventions to enhance vaccination coverage and mitigate Mpox transmission.

The current Mpox outbreak continues to primarily affect MSM and those who report recent sexual contact with one or more male partners [40]. Currently, three vaccines are approved for the prevention of Mpox: the JYNNEOS vaccine from Denmark, the ACAM2000 vaccine from the United States, and the LC16m8 vaccine from Japan [14]. Vaccination provides cross protection against smallpox and Mpox, preventing about 85% of Mpox virus infections [17]. A systematic review and meta-analysis encompassing 11 studies revealed a Mpox vaccine acceptance rate of 56%, indicating moderate acceptance. Notably, the acceptance rate was higher among the LGBTI community (84%) compared to the general population (43%), who often perceive the disease as restricted to the LGBTI community, accompanied by associated stigmatization [41]. Another study by Riad A et al. found that 51% of respondents were willing to receive the Mpox vaccinations if they were free and effective [42]. Additionally, a systematic review and meta-analysis including 29 studies determined a pooled prevalence of Mpox vaccination intention at 61% [17]. The Chinese population subgroup exhibited a significantly higher intention rate of 90.2% [43], surpassing the global average. This study found a high willingness to vaccinate among 87.1% of participants, showing similar results. This disparity may be attributed to the varying severity of Mpox outbreaks, preventive measures, and cultural factors across countries, along with differences in information access and trust in medical systems.

Our study is the first to develop the Mpox vaccine hesitancy scale, which is particularly significant in this context. It provides a standardized method for measuring vaccine hesitancy among MSM, a crucial step towards understanding and addressing the factors contributing to vaccine reluctance in this population. Grounded in PMT, this scale assesses cognitive processes that influence individuals’ decisions about vaccination. PMT posits that protection motivation arises from the evaluation of both threat and coping appraisals. By integrating PMT into the scale design, we finally formulated four dimensions: Maladaptive Rewards, Self-efficacy, Response Efficacy, and Response Costs.

The Chinese version of the Mpox vaccine hesitancy scale demonstrated excellent psychometric properties through comprehensive item analysis, ensuring high item discrimination with each item showing a strong correlation to the scale. The Cronbach’s α coefficient in the item analysis ranged from 0.904 to 0.915, indicating robust internal consistency even with the removal of individual items. These findings support the retention of all items in the scale.

Additionally, we thoroughly evaluated both the structural and content validity of the Mpox vaccine hesitancy scale. Structural validity assesses the alignment of the scale with theoretical assumptions, while content validity examines the extent to which the scale items fulfill the intended measurement objectives [33]. The scale displayed high content validity, with I-CVI scores ranging from 0.857 to 1.000 and S-CVI/Ave of 0.952, exceeding the reference value [44]. This indicates that the scale’s content is highly regarded by experts and suitable for MSM. EFA and CFA were used to assess the structural validity of the scale, revealing a clear scale with a final 22 items and a well-fitting structural equation model. Convergent validity, indicated by CR values exceeding 0.70 and AVE values exceeding 0.50 for all dimensions [36], confirmed that items intended to measure the same construct were grouped together. Discriminant validity was established, as the square root of the AVE exceeded the correlation coefficients between the dimensions, indicating clear differentiation among them.

The reliability of the scale was examined using internal consistency and split-half reliability methods. A Cronbach’s α coefficient greater than 0.80 is considered excellent, from 0.60 to 0.80 is considered good, and less than 0.60 is thought to be not good [38]. Our Mpox vaccine hesitancy scale’s Cronbach’s α was 0.906, higher than the threshold, indicating excellent internal consistency. For the four dimensions, the Cronbach’s α ranged from 0.811 to 0.942. Furthermore, the split-half reliability coefficient was 0.756, with split-half reliability coefficients for the four dimensions ranging from 0.809 to 0.904, indicating acceptable split-half reliability. The ROC analysis further demonstrated the scale’s strong predictive ability to effectively distinguish between different levels of vaccine hesitancy, highlighting its potential applicability in various settings.

In addition, our findings also contribute to the growing body of evidence on Mpox vaccine hesitancy by providing insights specific to the MSM population in China. A study by Gallè et al., which evaluated Mpox-related knowledge and vaccine acceptance among the general Italian population, reported a high degree of vaccine hesitancy, primarily attributed to a lack of confidence in vaccines (38.5%) and insufficient understanding of the severity of Mpox (37.7%) [45]. Similarly, in our study, “Response Costs”, which includes concerns about vaccine efficacy, and “Maladaptive Rewards”, which encompasses multiple forms of lack of understanding of Mpox severity (see Supplementary Table S2), emerged as key factors influencing hesitancy within this population. Furthermore, we observed connections between Mpox vaccine hesitancy and both Self-efficacy and Response Efficacy, offering a broader and more systematic understanding of the factors contributing to vaccine hesitancy. These findings underscore the need for targeted intervention strategies that address not only informational gaps but also practical concerns unique to marginalized populations. By focusing on these response costs, public health initiatives can more effectively reduce vaccine hesitancy and increase vaccination uptake among MSM communities.

Several limitations should be acknowledged. First, our participants were recruited between October 2023 and March 2024. While this study focused on MSM due to the nature of the 2022 Mpox outbreak, the evolving significance of non-sexual transmission in the August 2024 outbreak [8] suggests that our findings may not fully capture the broader dynamics of Mpox transmission and vaccine hesitancy in different contexts. Furthermore, the study sample was limited to Chinese, which may further impact the generalizability of the results to other populations and cultural settings. Second, the snowball sampling method and the unique characteristics of the MSM population may have introduced selection bias, as it likely excluded individuals with limited access to others. The internet-based data collection method may have also introduced selection bias, as it likely favored individuals with higher levels of education and propensity to share some information over the internet. Third, the cross-sectional design of the study limits the ability to establish causal relationships between the identified factors and vaccine hesitancy. Lastly, the self-reported nature of the data may be subject to social desirability bias, potentially leading participants to underreport their hesitancy or overreport their willingness to vaccinate. Future research should address these limitations by including diverse populations and utilizing mixed-method approaches to gain a more comprehensive understanding of Mpox vaccine hesitancy.

In summary, the Mpox vaccine hesitancy scale, grounded in PMT, is a reliable and valid tool for assessing vaccine hesitancy among MSM in China. Its strong psychometric properties ensure accurate measurement of cognitive and motivational factors influencing vaccination decisions. By identifying key dimensions such as Maladaptive Rewards, Self-efficacy, Response Efficacy, and Response Costs, the scale provides valuable insights for targeted interventions and public health strategies. These efforts may enhance vaccination uptake and control Mpox outbreaks among high-risk populations. Future research should explore the scale’s applicability in other cultural contexts to confirm its global relevance.

## 5. Conclusions

The Mpox vaccine hesitancy scale, grounded in PMT, provides a valid and reliable tool for assessing the vaccine intention among MSM in China. Our findings revealed distinct dimensions of vaccine hesitancy, such as Maladaptive Rewards, Self-efficacy, Response Efficacy, and Response Costs, offering valuable insights for targeted public health interventions. Despite limitations such as the study’s cross-sectional design and potential selection bias, the scale’s strong validity supports its use in understanding cognitive and motivational factors influencing vaccination decisions. Future research should validate the scale across diverse populations and cultural contexts to enhance its global applicability.

## Figures and Tables

**Table 1:** Basic population characteristics for all included men who have sex with men in 6 cities of China (N = 2403).

Characteristics	All Participants (N = 2403)	Low Vaccination Intention (N = 310)	High Vaccination Intention (N = 2093)	*p*-Value
Demographic characteristics				
Age, years [Mean ± SD]	30.59 ± 8.02	31.98 ± 8.58	30.38 ± 7.91	**0.002**
Education [n (%)]				0.054
Junior high school and below	157 (6.5)	30 (9.7)	127 (6.1)	
Senior high school	358 (14.9)	43 (13.9)	315 (15.1)	
Secondary vocational school and above	1888 (78.6)	237 (76.5)	1651 (78.9)	
Residence [n (%)]				**<0.001**
Shanghai	569 (23.7)	58 (18.7)	511 (24.4)	
Guangdong	500 (20.8)	61 (18.7)	439 (21.0)	
Liaoning	502 (20.9)	98 (31.6)	404 (19.3)	
Shaanxi	199 (8.3)	19 (6.1)	180 (8.6)	
Yunnan	313 (13.0)	52 (16.8)	261 (12.5)	
Xinjiang	320 (13.3)	22 (7.1)	198 (14.2)	
Marriage [n (%)]				**0.012**
Unmarried	2035 (84.7)	245 (79.0)	1790 (85.5)	
Married	255 (10.6)	46 (14.8)	209 (10.0)	
Divorced and widowed	113 (4.7)	19 (6.1)	94 (4.5)	
Occupation [n (%)]				0.084
Employed	1160 (48.3)	154 (49.7)	1006 (48.1)	
Unemployed	923 (38.4)	127 (41.0)	796 (38.0)	
Student	320 (13.3)	29 (9.4)	291 (13.9)	
Monthly income, CNY [n (%)]				**0.017**
≤3000	502 (20.9)	66 (21.3)	436 (20.8)	
3001–6000	851 (35.4)	128 (41.3)	723 (34.5)	
6001–12,000	802 (33.4)	80 (25.8)	722 (34.5)	
≥12,001	248 (10.3)	36 (11.6)	212 (10.1)	
Sex related				
Homosexual orientation only [n (%)]				**0.001**
No	516 (21.5)	90 (29.0)	426 (20.4)	
Yes	1887 (78.5)	220 (71.0)	1667 (79.6)	
HIV status [n (%)]				0.462
Negative or unknown	2204 (91.7)	281 (90.6)	1923 (91.9)	
Positive	199 (8.3)	29 (9.4)	170 (8.1)	
Sexually transmitted diseases [n (%)]				**0.004**
No	2182 (90.8)	295 (95.2)	1887 (90.2)	
Yes	221 (9.2)	15 (4.8)	206 (9.8)	
Number of sexual partners [n (%)]				0.509
Single	1206 (50.2)	161 (51.9)	1045 (49.9)	
Multiple	1197 (49.8)	149 (48.1)	1048 (50.1)	
Unprotected anal intercourse [n (%)]				0.391
Never or sometimes	1395 (58.1)	173 (55.8)	1222 (58.4)	
Always	1008 (41.9)	137 (44.2)	871 (41.6)	
Lifestyles				
Smoking [n (%)]	939 (39.1)	117 (37.7)	822 (39.3)	0.606
Drinking [n (%)]	1281 (53.5)	166 (53.5)	1115 (53.3)	0.928
Disease history				
Hypertension [n (%)]	155 (6.5)	30 (9.7)	125 (6.0)	**0.013**
Dyslipidemia [n (%)]	222 (9.2)	36 (11.6)	186 (8.9)	0.122
Diabetes [n (%)]	69 (2.9)	9 (2.9)	60 (2.9)	0.971
Mpox related				
Mpox diagnosis [n (%)]	56 (2.3)	5 (1.6)	51 (2.4)	0.370

Data are presented as mean ± SD for continuous variables and number (%) for categorical variables. The independent samples *t*-test and chi-square test were conducted for comparisons between groups for continuous variables and categorical variables, respectively. Bold *p*-values indicate *p* < 0.05.

**Table 2:** Descriptive statistics and item analysis of the Chinese vaccination intention scale for men who have sex with men (N = 2403).

Items	Score Range	Mean ± SD	Absolute Value of Critical Ratio (CR)	Item–Total Correlation (r)	Cronbach’s α with Item Deleted
Maladaptive Rewards	7–35	16.98 ± 6.53			
Q1		2.40 ± 1.06	47.454 ***	0.891 ***	0.905
Q2		2.40 ± 1.09	45.493 ***	0.873 ***	0.905
Q3		2.38 ± 1.06	47.010 ***	0.905 ***	0.904
Q4		2.37 ± 1.06	49.141 ***	0.898 ***	0.904
Q5		2.44 ± 1.07	42.245 ***	0.851 ***	0.905
Q6		2.48 ± 1.13	36.962 ***	0784 ***	0.907
Q7		2.52 ± 1.14	41.207 ***	0.816 ***	0.906
Self-efficacy	7–35	16.54 ± 5.61			
Q8		2.33 ± 0.92	33.348 ***	0.806 ***	0.907
Q9		2.51 ± 0.97	26.910 ***	0.783 ***	0.908
Q10		2.38 ± 0.92	32.529 ***	0.856 ***	0.907
Q11		2.34 ± 0.93	36.888 ***	0.903 ***	0.906
Q12		2.33 ± 0.93	38.035 ***	0.898 ***	0.906
Q13		2.34 ± 0.93	37.387 ***	0.897 ***	0.906
Q14		2.30 ± 0.92	37.917 ***	0.896 ***	0.906
Response Efficacy	4–20	8.37 ± 3.24			
Q15		2.18 ± 1.05	16.195 ***	0.810 ***	0.913
Q16		2.11 ± 0.92	19.468 ***	0.895 ***	0.912
Q17		2.00 ± 0.89	20.148 ***	0.890 ***	0.911
Q18		2.09 ± 0.90	21.541 ***	0.877 ***	0.911
Response Costs	6–30	18.24 ± 4.82			
Q19		2.95 ± 1.08	24.681 ***	0.637 ***	0.909
Q20		2.63 ± 1.05	32.889 ***	0.702 ***	0.908
Q21		3.38 ± 1.14	11.123 ***	0.692 ***	0.915
Q22		3.31 ± 1.10	19.752 ***	0.823 ***	0.911
Q23		3.24 ± 1.10	16.958 ***	0.805 ***	0.912
Q24		2.74 ± 1.14	22.699 ***	0.710 ***	0.911

*** indicates *p* < 0.001.

**Table 3:** Factor loadings after rotation of the Chinese Mpox vaccination intention scale for men who have sex with men (N = 1202).

Items	Standardized Factor Loadings			
	Factor 1 Maladaptive Rewards	Factor 2 Self-Efficacy	Factor 3 Response Efficacy	Factor 4 Response Costs
F1-1 (Q1)	**0.868**	0.218	0.029	0.098
F1-2 (Q2)	**0.838**	0.224	0.019	0.137
F1-3 (Q3)	**0.897**	0.181	0.039	0.097
F1-4 (Q4)	**0.874**	0.191	0.059	0.115
F1-5 (Q5)	**0.811**	0.175	0.028	0.126
F1-6 (Q6)	**0.731**	0.135	−0.014	0.173
F1-7 (Q7)	**0.763**	0.183	−0.040	0.164
F2-1 (Q8)	0.195	**0.719**	0.315	−0.093
F2-2 (Q9)	0.093	**0.725**	0.215	0.018
F2-3 (Q10)	0.163	**0.778**	0.277	−0.083
F2-4 (Q11)	0.240	**0.877**	0.142	0.018
F2-5 (Q12)	0.241	**0.876**	0.136	0.045
F2-6 (Q13)	0.221	**0.879**	0.134	0.046
F2-7 (Q14)	0.266	**0.862**	0.150	0.041
F3-1 (Q15)	−0.028	0.251	**0.728**	−0.068
F3-2 (Q16)	−0.002	0.222	**0.881**	−0.005
F3-3 (Q17)	0.022	0.225	**0.873**	−0.065
F3-4 (Q18)	0.053	0.259	**0.852**	−0.067
F4-1 (Q21)	0.052	0.018	−0.183	**0.735**
F4-2 (Q22)	0.223	0.040	−0.079	**0.849**
F4-3 (Q23)	0.176	0.013	−0.096	**0.869**
F4-4 (Q24)	0.267	-0.094	0.217	**0.661**
Explained variance (%)	23.933	23.597	14.536	11.878

Bold indicates the highest factor loadings for each item, suggesting the primary factor on which the item loads most strongly.

**Table 4:** Model fit index of the Chinese Mpox vaccination intention scale for men who have sex with men (N = 1201).

Model Fitting Index	Reference Value	Model Value
χ^2^/df	<5.000	4.382
RMSEA	<0.080	0.053
SRMR	<0.050	0.048
GFI	>0.900	0.935
CFI	>0.900	0.967
NFI	>0.900	0.958
TLI	>0.900	0.963
IFI	>0.900	0.967

*χ*^2^/df: Chi-square goodness-of-fit; RMSEA: root mean square error of approximation; SRMR: standardized root mean square residual; GFI: goodness-of-fit index; CFI: comparative fit index; NFI: normed fit index; TLI: Tucker–Lewis index; IFI: incremental fit index.

**Table 5:** Convergent validity and discriminant validity of the Chinese Mpox vaccination intention scale for men who have sex with men (N = 1201).

Subscale	Combined Reliability (CR)	Average Variance Extracted (AVE)	Maladaptive Rewards	Self-Efficacy	Response Efficacy	Response Costs
Maladaptive Rewards	0.940	0.693	**0.832**	—	—	—
Self-efficacy	0.940	0.695	0.402 ***	**0.834**	—	—
Response Efficacy	0.889	0.670	0.141 ***	0.492 ***	**0.819**	—
Response Costs	0.821	0.543	0.403 ***	0.118 ***	−0.030 ***	**0.737**

The diagonal bold numbers are AVE square root values; other numbers are inter-construct correlation coefficients; *** indicates significance at *p* < 0.001.

## Data Availability

The data presented in this study are available upon request from the corresponding author. Due to privacy and ethical restrictions, the data are not publicly available to protect the participants’ privacy.

## Supplementary Materials

The following supporting information can be downloaded at https://www.mdpi.com/article/10.3390/vaccines12091009/s1, Table S1: Factor loadings after rotation of the Chinese Mpox vaccination intention scale for men who have sex with men (N = 1202); Table S2: The 22-item Mpox vaccination hesitation scale for men who have sex with men; Table S3: Convergent validity of the Chinese Mpox vaccination intention scale for men who have sex with men (N = 1201); Table S4: Results of the reliability evaluation (N = 2403).
