# Supplementary material for: The Mpox Vaccine Hesitancy Scale for Mpox: Links with Vaccination Intention among Men Who Have Sex with Men in Six Cities of China
Source: Vaccines (Basel). 2024 Sep 3;12(9):1009. doi: 10.3390/vaccines12091009 (PMC11436122; doi:10.3390/vaccines12091009)
Supplement: Supplementary file 1 [file vaccines-12-01009-s001.zip › vaccines-3112925-supplementary.pdf]

Supplementary Materials

Supplementary Table S1. Factor loadings after rotation of the Chinese Mpox vaccination intention scale for men who have sex with men (N = 1,202).

Items	Standardized Factor Loadings			
	Factor 1 Maladaptive Rewards	Factor 2 Self-efficacy	Factor 3 Response Efficacy	Factor 4 Response Costs
Q1	**0.867**	0.211	0.037	0.104
Q2	**0.835**	0.216	0.030	0.138
Q3	**0.896**	0.174	0.049	0.105
Q4	**0.872**	0.184	0.069	0.117
Q5	**0.810**	0.168	0.038	0.131
Q6	**0.728**	0.126	-0.003	0.174
Q7	**0.762**	0.176	-0.031	0.172
Q8	0.195	**0.720**	0.313	-0.090
Q9	0.097	**0.731**	0.201	0.032
Q10	0.164	**0.781**	0.270	-0.076
Q11	0.242	**0.876**	0.141	0.034
Q12	0.243	**0.873**	0.136	0.060
Q13	0.223	**0.876**	0.134	0.062
Q14	0.266	**0.857**	0.153	0.049
Q15	-0.029	0.264	**0.715**	-0.061
Q16	-0.008	0.229	**0.877**	-0.009
Q17	0.017	0.233	**0.870**	-0.065
Q18	0.046	0.265	**0.852**	-0.072
Q19 (deleted)	**0.457**	0.202	-0.231	0.355
Q20 (deleted)	0.478	0.217	-0.058	**0.493**
Q21	0.048	0.004	-0.174	**0.742**
Q22	0.214	0.018	-0.059	**0.835**
Q23	0.167	-0.009	-0.078	**0.858**
Q24	0.260	-0.106	0.227	**0.654**
Explained variance(%)	23.659	21.955	13.390	12.293

Supplementary Table S2. The 22-item Mpox vaccination hesitation scale for men who have sex with men.

Scale items
**Maladaptive Rewards**
F1-1. I believe that I am very healthy, so I do not need the Mpox vaccine.
F1-2. I think I have no history of unclean sexual activities, so I do not need the Mpox vaccine.
F1-3. I have previously undergone relevant tests and the results were normal, so I do not need the Mpox vaccine.
F1-4. People around me have not been vaccinated against Mpox and have not contracted the related disease, so I do not need the Mpox vaccine either.
F1-5. My friends believe that if there are no physical discomforts, there is no need to get the Mpox vaccine.
F1-6. If I get the Mpox vaccine, people might think I have a history of unclean sexual activities, which would negatively affect my interpersonal relationships.
F1-7. My friends do not intend to get the Mpox vaccine, so if I get vaccinated, it would seem unusual.
**Self-efficacy**
F2-1. Even if my physical examination results are normal, I would still get the Mpox vaccine.
F2-2. Even if the cost of the Mpox vaccine is not covered by insurance and requires out-of-pocket payment, I would still get vaccinated.
F2-3. Even if my family and friends think that getting the Mpox vaccine is unnecessary, I would still get vaccinated.
F2-4. Even if the vaccination site is far away, I would follow the vaccination schedule and get the Mpox vaccine.
F2-5. Even if I am always busy with work, I would follow the vaccination schedule and get the Mpox vaccine.
F2-6. Even if it requires taking time off from work to get vaccinated, I would still get the Mpox vaccine.
F2-7. Even if none of my friends have received the Mpox vaccine, I would still get vaccinated.
**Response Efficacy**
F3-1. Getting the Mpox vaccine can provide more health benefits.
F3-2. Getting the Mpox vaccine can effectively prevent Mpox infection.
F3-3. Getting the Mpox vaccine will reduce the risk of contracting Mpox.
F3-4. Getting the Mpox vaccine allows me to fully engage in my studies, work, and life.
**Response Costs**
F4-1. I do not know where to get the Mpox vaccine.
F4-2. I am worried that the vaccine is not safe and that there may be side effects after vaccination.
F4-3. I am concerned that the vaccine might not be effective.
F4-4. I am worried that getting the Mpox vaccine might actually result in contracting Mpox.

Supplementary Table S3. Convergent validity of the Chinese Mpox vaccination intention scale for men who have sex with men (N = 1,201).

Subscale	Items	Unstandardized factor loading	Standardized factor loading	Combined reliability (CR)	Average variance extracted (AVE)
Maladaptive Rewards	F1-1 (Q1)	1.000	0.903	0.940	0.693
	F1-2 (Q2)	0.986	0.885		
	F1-3 (Q3)	1.017	0.917		
	F1-4 (Q4)	1.012	0.917		
	F1-5 (Q5)	0.904	0.819		
	F1-6 (Q6)	0.759	0.652		
	F1-7 (Q7)	0.810	0.689		
Self-efficacy	F2-1 (Q8)	1.000	0.736	0.940	0.695
	F2-2 (Q9)	0.987	0.704		
	F2-3 (Q10)	1.069	0.803		
	F2-4 (Q11)	1.172	0.895		
	F2-5 (Q12)	1.198	0.899		
	F2-6 (Q13)	1.198	0.896		
	F2-7 (Q14)	1.166	0.879		
Response Efficacy	F3-1 (Q15)	1.000	0.68	0.889	0.670
	F3-2 (Q16)	1.171	0.879		
	F3-3 (Q17)	1.105	0.863		
	F3-4 (Q18)	1.077	0.835		
Response Costs	F4-1 (Q21)	1.000	0.573	0.821	0.543
	F4-2 (Q22)	1.499	0.875		
	F4-3 (Q23)	1.412	0.828		
	F4-4 (Q24)	1.136	0.625		

Supplementary Table S4. Results of the reliability evaluation (N = 2,403).

Subscale	Number of items	Cronbach's α	Split-half reliability
Maladaptive Rewards	7	0.941	0.848
Self-efficacy	7	0.942	0.904
Response Efficacy	4	0.887	0.886
Response Costs	4	0.811	0.809
Total	22	0.906	0.756
